# Harnessing Metabolic Priming to Engineer Human Nucleus Pulposus Macromass Overcoming Scalability‐Phenotype Tradeoff

**DOI:** 10.1111/cpr.70215

**Published:** 2026-04-28

**Authors:** Yingbo Wang, Ou Hu, Jian Wu, Sha Huang, Peng Lin, Pulin Yan, Yangyang Li, Huaijian Jin, Yutong Wu, Jun Zhu, Jungang Pu, Bo Hu, Jian He, Yibo Gan, Peng Liu

**Affiliations:** ^1^ Department of Spine Surgery, Center of Orthopedics, State Key Laboratory of Trauma and Chemical Poisoning, Chongqing Key Laboratory of Spinal Disease Therapy and Regeneration (Military‐Civilian) Daping Hospital, Army Medical University Chongqing China; ^2^ Department of Orthopedics Bishan Hospital, Chongqing University of Chinese Medicine Chongqing China; ^3^ Tissue Stress Injury and Functional Repair Key Laboratory of Sichuan Province & Preclinical Research Center The General Hospital of Western Theater Command Chengdu China

**Keywords:** extracellular matrix, fatty acid oxidation, intervertebral disc, mitochondria, nucleus pulposus

## Abstract

Intervertebral disc degeneration (IDD) represents a major global health challenge, primarily due to the inability of current therapies to reverse the progressive loss of nucleus pulposus (NP) tissue and function. Transplanting bioactive substitutes offers potential for overcoming this limitation. However, current strategies fail to generate large‐sized, functional human NP substitutes, impeded by a fundamental trade‐off: the incapacity to simultaneously achieve scalable expansion and maintain the essential cellular phenotype. Here, we found that human platelet lysate (hPL) acts not merely as a growth supplement but as a powerful metabolic primer, driving robust proliferation of human NP cells (hNPCs) while remarkably preserving a mature NP phenotype. This was demonstrated through sustained expression of aggrecan (ACAN) and collagen type II (COL2). Crucially, this metabolic shift allowed us to create a high‐quality, homogeneous NP macromass exceeding 2 mm in size, which exhibited superior mechanical integrity and successfully avoided the common problem of necrotic core formation. In vivo validation demonstrated significantly larger grafts with markedly enhanced ACAN and COL2 deposition, confirming the functional superiority of the constructs. Mechanistically, transcriptomic analysis revealed that hPL specifically enhanced fatty acid oxidation (FAO), with this energy metabolism shift serving as the primary driver enabling both rapid growth and phenotypic stability. Ultimately, the hPL‐primed NP macromass demonstrated exceptional efficacy in repairing degenerated discs in situ. Our work introduces a novel and potent paradigm for IDD treatment by harnessing the principle of metabolic priming to generate scalable and functional NP substitutes, effectively bridging a critical gap in disc regeneration therapy.

## Graphical abstract

Human platelet lysate acts as a metabolic primer for hNPCs, enhancing fatty acid oxidation to drive proliferation while preserving phenotype. This enables construction of high‐quality, large‐size macromasses without necrotic defects, demonstrating exceptional efficacy in repairing degenerated discs in situ and providing a novel paradigm for intervertebral disc regeneration therapy.

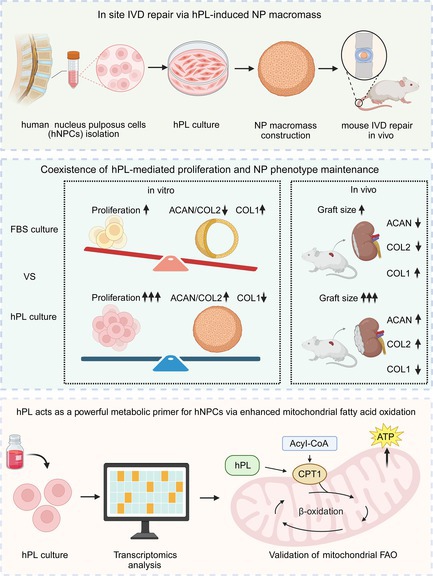

## 1 Introduction

Low back pain (LBP) causes chronic disability worldwide and imposes a heavy healthcare burden [1]. Intervertebral disc degeneration (IDD) is widely recognized as a primary contributor to LBP, characterized by the progressive deterioration of the structural integrity and biological function of the intervertebral disc (IVD) [2, 3, 4]. The nucleus pulposus (NP) maintains disc homeostasis by absorbing shock and distributing mechanical loads [5]. However, the NP exhibits severely limited self‐healing capacity due to its harsh microenvironment characterized by hypoxia, low pH, high osmotic pressure and restricted nutrient diffusion [6, 7]. Developing effective therapies for functional NP regeneration therefore remains an urgent priority.

Human nucleus pulposus cells (hNPCs) are the primary cells responsible for synthesizing and maintaining the extracellular matrix (ECM) of the NP, which primarily comprises aggrecan (ACAN) and collagen type II (COL2) [8]. The construction of three‐dimensional (3D) NP macromass that reproduces native tissue structure and biological function represents an essential advance toward hNPC clinical translation. However, current strategies face fundamental limitations in generating sufficient substitutes, as engineered tissues cannot simultaneously achieve robust cell proliferation and stable NP cellular phenotype [9, 10]. hNPCs are scarce and proliferate poorly in vitro [11], and conventional culture media cause phenotypic drift with decreased expression of NP‐specific proteins (ACAN, COL2) and increased collagen type I (COL1) [12, 13]. Moreover, when 3D constructs exceed 1.5 mm in diameter using current methodologies, central regions often develop necrotic cores due to nutrient and oxygen diffusion constraints, resulting in heterogeneous tissue quality [10, 14, 15]. Therefore, new strategies are needed to overcome these challenges.

Human platelet lysate (hPL) is a xeno‐free alternative that supports cell expansion, maintains stem cell properties and has recently been shown to enhance mitochondrial function [16, 17, 18, 19, 20, 21]. However, the majority of studies on hPL have been conducted in monolayer culture systems, and its application in 3D culture platforms remains limited to a few scaffold‐based approaches [22, 23]. Whether hPL can simultaneously drive sufficient hNPC expansion and stabilize NP‐specific phenotype within scaffold‐free 3D constructs, thereby providing a viable strategy for fabricating functional NP substitutes, has not been investigated. In this study, we show that hPL serves as a powerful metabolic primer, simultaneously driving robust hNPC proliferation and preserving their NP cellular phenotype. The hPL‐primed NP macromass has better mechanical properties and effectively repairs degenerated discs function in situ. We anticipate that this work provides a promising strategy and mechanistic basis for treating IDD.

## 2 Materials and Methods

### 2.1 Isolation and Culture of hNPCs

Human NP tissues were obtained from patients undergoing lumbar discectomy at Daping Hospital, Army Medical University, with approval from the Institutional Ethics Review Board (Ethics Committee [2023‐48]), and informed consent was obtained from all patients. All NP tissue samples were obtained from patients aged 30–50 years with Pfirrmann grade III disc degeneration, diagnosed with either contained lumbar disc herniation or lumbar spinal stenosis. Tissues were minced and enzymatically digested in DMEM/F12 (Gibco) containing 0.2% collagenase type II (Sigma), 0.2% pronase (Roche), 100 U/mL DNase I (Solarbio) and 0.01% hyaluronidase (Solarbio) at 37°C for 2–3 h. Isolated cells were filtered and cultured in DMEM/F12 supplemented with 10% or 20% fetal bovine serum (FBS, Gibco), or 5% hPL (EliteCell), each containing 1% penicillin–streptomycin (HyClone).

### 2.2 Cell Viability and Cell Proliferation Assay

For viability assessment, hNPCs were seeded in 96‐well plates at 3000 cells/well and cultured for 48 h in culture media containing either FBS or hPL. Cell viability was assessed using the Cell Counting Kit‐8 (CCK‐8, Dojindo) according to the manufacturer's instructions. Briefly, CCK‐8 solution was added to each well at a 1:10 (v/v) ratio, followed by incubation at 37°C for 2 h, and absorbance was recorded at 450 nm using a microplate reader (Thermo Fisher Scientific). For proliferation analysis, hNPCs were cultured under the respective conditions for 7 days. Proliferating cells were labelled using the Click‐iT EdU Kit (Invitrogen), and Ki67 expression was assessed by immunostaining with an anti‐Ki67 (D3B5) rabbit monoclonal antibody (Cell Signalling Technology) according to the respective manufacturers' protocols. Total cell numbers under different culture conditions were quantified using an automated cell counter (Countstar).

### 2.3 Cytoskeleton Visualization

Cells were fixed with 4% paraformaldehyde (PFA, Biosharp) for 30 min at room temperature, followed by permeabilization with 0.1% Triton X‐100 (Solarbio) for 10 min. After three washes with PBS (Gibco), cells were incubated with Phalloidin (Abcam) in the dark for 1 h at room temperature to visualize the cytoskeleton. Following an additional three PBS washes, nuclei were counterstained with 4′,6‐diamidino‐2‐phenylindole (DAPI, Beyotime). Fluorescence images were acquired using a fluorescence microscope (Olympus). Antibody information is detailed in Table S1.

### 2.4 3D Cell Culture in Scaffolds

Cells were resuspended in 5% gelatin methacrylate (GelMA) hydrogel (EFL) containing 0.05% lithium phenyl‐2,4,6‐trimethylbenzoylphosphinate photoinitiator (LAP, EFL), plated in 24‐well plates at a density of 3 × 10^5^ cells and crosslinked by exposure to 405 nm light for 15 s. After gelation, the medium was replaced every 72 h. To dissociate the cells from the 3D hydrogel culture for subsequent cell proliferation analysis, the hydrogel was digested using 0.1% collagenase type II dissolved in DMEM/F12 medium. Cell proliferation was then assessed using an automated cell counter on Day 7.

### 2.5 NP Macromass Construction

In this study, macromass is defined as a 3D scaffold‐free constructs exceeding 2 mm in diameter with structurally intact architecture and no evidence of central void. For NP macromass construction, 5 × 10^5^ hPL‐hNPCs were resuspended in 1 mL of chondrogenic induction medium (Chondrogenesis Differentiation Kit, STEMCELL Technologies) in a 15 mL conical tube and centrifuged at 300 × *g* for 5 min to form aggregates. The tube cap was loosened for gas exchange. On Day 3, 0.5 mL of fresh medium was added. From Day 6 onward, the medium was carefully replaced with 1 mL of fresh induction medium every 3 days until Day 28.

### 2.6 Histological Staining

At Day 28, pellets derived from hNPCs were harvested and fixed in 4% PFA. For IVD specimens, harvested samples were similarly fixed in 4% PFA and subsequently subjected to decalcification prior to further processing. All specimens were then paraffin‐embedded and sectioned. Macromass sections were stained with Alcian blue (Sigma‐Aldrich) and Safranin‐O/fast green (SO/FG, Solarbio), whereas IVD sections underwent haematoxylin and eosin (H&E, BBI) and SO/FG. All sections were mounted with neutral balsam and examined under light microscopy. Histological grading of IVD sections was performed according to the scoring system described by Tam et al., with all sections evaluated independently by two blinded observers.

### 2.7 Immunofluorescence Staining

Paraffin‐embedded sections were deparaffinized in xylene and rehydrated through a graded ethanol series, followed by antigen retrieval. After PBS washes, sections were permeabilized with 0.3% Triton X‐100 for 15 min and blocked with 0.5% BSA in PBS for 30 min at room temperature. Primary antibodies were then applied and incubated overnight at 4°C. Following thorough PBS rinsing, sections were incubated with secondary antibodies for 1 h at room temperature in the dark. Finally, sections were counterstained with DAPI, mounted and visualized under a fluorescence microscope to assess target protein expression (see Table S1 for antibody information).

### 2.8 Kidney Capsule Implantation

All animal experiments were conducted in accordance with institutional guidelines and approved by the Animal Ethics Committee. Briefly, cells were resuspended in Matrigel (Corning) at 5 × 10^5^ cells/5 μL. Six‐week‐old NPG mice (Vital River Laboratories, *n* = 5 per group) were anaesthetised by intraperitoneal injection of pentobarbital sodium (80 mg kg^−1^). Under sterile conditions, a small flank incision was made to expose the kidney, and the cell suspension was injected into the subrenal capsular space. The incision was closed with absorbable sutures, and mice were placed on a heating pad until recovery. After 8 weeks, the grafts were removed for analysis.

### 2.9 Movat Pentachrome Stain

Movat pentachrome staining (ScyTek) was performed on 4% PFA‐fixed, paraffin‐embedded sections following the manufacturer's protocol. Sections were sequentially stained with elastic stain solution (20 min), differentiated in ferric chloride, treated with sodium thiosulfate (1 min), stained with Alcian blue (pH 2.5, 15–30 min) and subjected to trichrome staining. Sections were then dehydrated and mounted.

### 2.10 Bulk RNA Sequencing and Analysis

Cell suspensions from cultures supplemented with 10% FBS or 5% hPL underwent enzymatic dissociation. Total RNA was extracted using TRIzol reagent (BBI) following the standard protocol, with subsequent verification of purity and integrity. Libraries were constructed and RNA sequencing was conducted by BGI (China) on the DNBSEQ platform. Read quality was evaluated using FastQC, demonstrating excellent data. Clean reads were aligned to the reference genome via HISAT2 (v2.2.1). Bowtie2 (v2.3.4.13) then mapped these reads against the gene set, encompassing both known and novel transcripts alongside coding and noncoding sequences. Gene expression levels were quantified using RSEM (v1.3.1). Differentially expressed genes were identified using DESeq2 (v1.4.5) based on | log_2_(fold change) | ≥ 1 and adjusted *p*‐value ≤ 0.05. Heatmaps displaying gene expression variation across samples were generated using Pheatmap (v1.0.8). Phenotypic alterations were investigated through GO (http://www.geneontology.org/) and KEGG (https://www.kegg.jp/) enrichment analyses of annotated differentially expressed genes via hypergeometric testing. Adjusted *p*‐values were used to correct for multiple testing, with significance set at *p* ≤ 0.05.

### 2.11 ATP Production Assay

Intracellular ATP was assessed with the ATP Assay Kit (MedChemExpress). Following lysis on ice, cell lysates underwent centrifugation at 12,000 × *g* for 5 min at 4°C. ATP content in the resulting supernatants was quantified through luminescence detection on a microplate reader per the manufacturer's instructions. ATP concentrations were calculated from a standard curve.

### 2.12 Pharmacological Inhibition Assays

Etomoxir and Compound C (both MedChemExpress) were dissolved in DMSO to stock concentrations of 20 and 10 mM, respectively, and stored at −20°C. Working solutions were freshly diluted in culture medium immediately before use. hNPCs in 5% hPL‐supplemented medium were treated with 40 μM etomoxir or 10 μM Compound C.

### 2.13 Atomic Force Microscopy

Freshly formed 3D tissue cultured in hPL or FBS medium was embedded in OCT and cryosectioned at a thickness of 50 μm. Sections were fixed on the slides and kept at 4°C to avoid dehydration. Atomic force microscopy (AFM) measurements were performed on a Dimension Icon Scanasyst with a Nanoscope 6 controller (Bruker). For the indentation test, cantilevers coated with reflective gold with a symmetric tip (radius = 20 nm) were used.

### 2.14 Magnetic Resonance Imaging (MRI)

At 12 weeks post‐surgery, mice (*n* = 5 per group) were anaesthetised (80 mg kg^−1^) and positioned in a 7.0T MRI system (Bruker) for disc imaging. A TurboRARE sequence was used to capture high‐resolution T2‐weighted images of caudal intervertebral discs. Disc degeneration was evaluated by quantifying NP signal intensity from mid‐sagittal images.

### 2.15 Implantation of NP Macromass in Mouse Caudal Intervertebral Discs

Male NPG mice aged 4–5 weeks (Vital River Laboratories) were randomly divided into three groups: control group (sham surgery), hNPCs transplantation group (2D cells) and macromass transplantation group (3D tissue). After intraperitoneal anaesthesia with pentobarbital sodium, caudal intervertebral discs were surgically exposed under microscopy and annular puncture was performed using a 30G needle to induce NP extrusion. 2D cells or macromass were engrafted into punctured discs, while control mice underwent only skin incision and suturing without disc puncture or cell transplantation. All animals were maintained for 4, 8 and 12 weeks postoperatively before subsequent analyses.

### 2.16 Western Blot Analysis

Total protein was extracted from cells using RIPA lysis buffer (Beyotime). For electrophoresis, the protein sample was loaded onto SDS‐PAGE gels, with subsequent transfer to PVDF membranes (Beyotime). For non‐phosphorylated targets, membranes were blocked with 5% non‐fat milk in Tris‐buffered saline with Tween 20 (TBST), while 5% BSA in TBST was used for phosphorylated targets. Membranes were then incubated with primary antibodies overnight at 4°C, washed three times with TBST and incubated with HRP‐conjugated secondary antibodies for 1 h at room temperature. Bands were visualized by ECL (Thermo Fisher) and quantified using ImageJ, with β‐actin as the loading control. Antibody details are listed in Table S1.

### 2.17 Statistical Analysis

All experiments were conducted with a minimum of three independent replicates. Data are presented as mean ± standard deviation (SD). Statistical comparisons between two groups were performed using unpaired two‐tailed Student's *t*‐tests. For comparisons involving multiple groups, one‐way ANOVA was applied followed by Tukey's test or Dunnett's multiple comparisons test where appropriate. A *p*‐value of less than 0.05 was considered statistically significant (n.s ≥ 0.05, *p < 0.05, **p < 0.01, ***p < 0.001, ****p < 0.0001). All statistical analyses were carried out using GraphPad Prism 9.0 software.

## 3 Results

### 3.1 hPL Robustly Enhances hNPCs Proliferation in 2D and 3D Culture Systems

**FIGURE 1:**
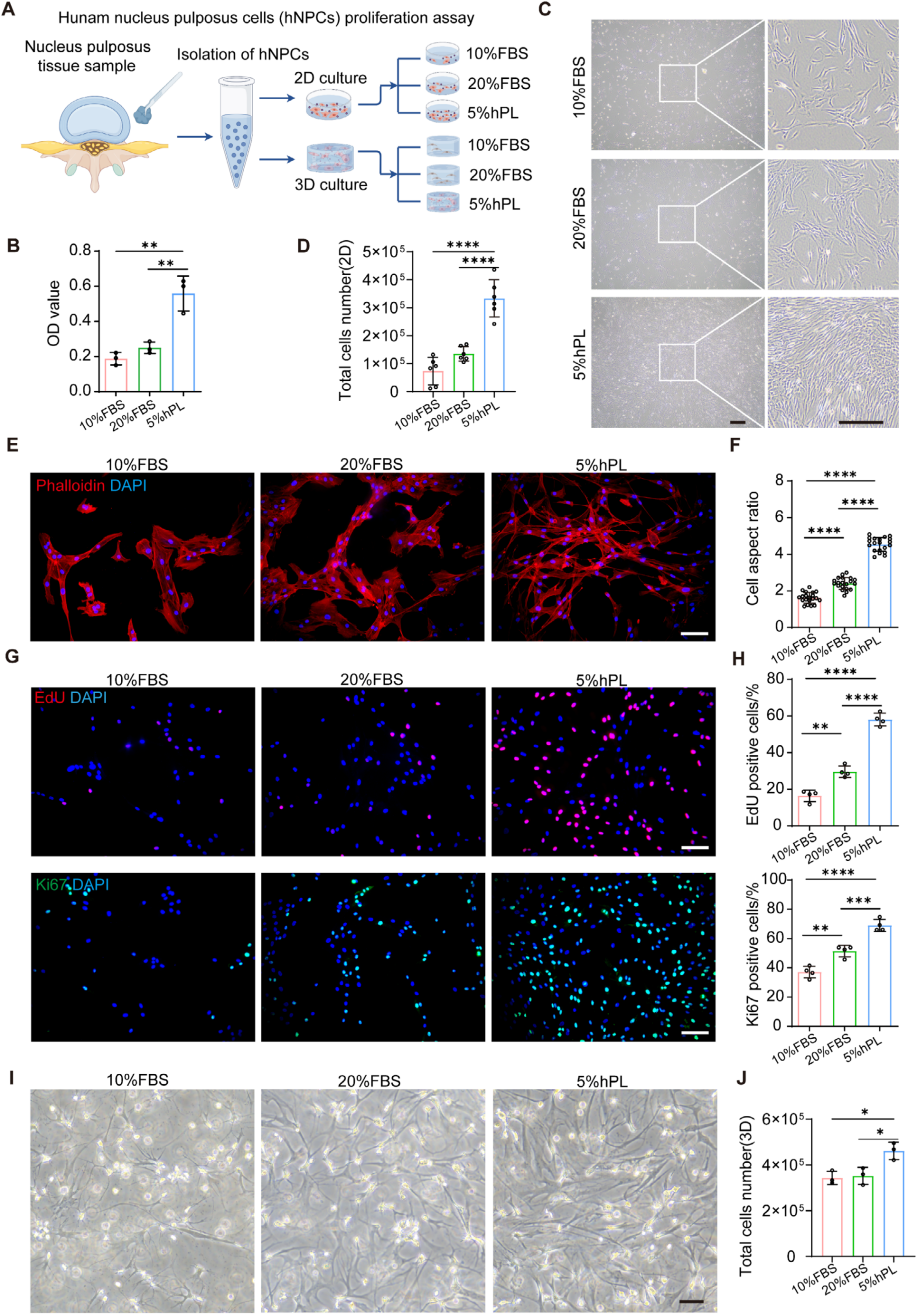
hPL culture promotes hNPC proliferation in both 2D and 3D culture systems. (A) Schematic of the experimental approach used to assess the proliferative effects of FBS and hPL on hNPCs. (B) Cell viability assessed by CCK‐8 assay (*n* = 3). (C) Representative brightfield images of hNPCs under different culture conditions. Scale bar: 250 μm. (D) Relative cell counts quantified using a cell counter (*n* = 6). (E) Representative phalloidin staining images. Scale bar: 100 μm. (F) Quantification of relative cell size (*n* = 20 cells/group). (G) Representative immunofluorescence images of EdU and Ki67 staining. Scale bar: 100 μm. (H) Quantification of EdU^+^ and Ki67^+^ cells (*n* = 4). (I) Representative images of hNPCs in 3D GelMA culture. Scale bar: 100 μm. (J) Quantification of cell counts from 3D cultures (*n* = 3). All data are presented as mean ± SD. *p < 0.05, **p < 0.01, ***p < 0.001, ****p < 0.0001, *p* values were determined using one‐way ANOVA followed by Dunnett's multiple comparison test.

To investigate the proliferative effects of hPL on hNPCs, cells were cultured under three medium conditions: DMEM supplemented with 10% FBS [24], 20% FBS [25], or 5% hPL (Figure 1A). The CCK‐8 assay revealed a higher absorbance in cells cultured under the hPL condition (hPL‐hNPCs) at Day 3 (Figure 1B), and morphological observation confirmed a visibly faster expansion compared to those in typical FBS conditions (FBS‐hNPCs) (Figure 1C). Direct cell counting after 7 days demonstrated that hPL‐hNPCs expanded 2.46‐fold more than 20% FBS‐hNPCs and 4.55‐fold more than 10% FBS‐hNPCs (Figure 1D). Phalloidin staining further revealed reduced cell size in hPL‐hNPCs, consistent with their actively proliferative phenotype [26] (Figure 1E,F).

To directly quantify proliferation, we performed immunostaining for proliferation markers Ki67 and 5‐ethynyl‐2′‐deoxyuridine (EdU). Both markers demonstrated a significantly higher proportion of proliferative cells in hPL‐hNPCs compared to both 20% FBS‐hNPCs and 10% FBS‐hNPCs (Ki67: 68.96% vs. 51.48% vs. 37.01%; EdU: 58.12% vs. 29.62% vs. 16.39%, respectively) (Figure 1G,H). To determine whether these effects persist in a 3D environment, hNPCs were encapsulated in GelMA hydrogel scaffolds and cultured for 7 days, after which cells were recovered by collagenase type II digestion and counted. Consistent with 2D results, hPL‐hNPCs exhibited enhanced proliferation under 3D conditions, confirming that this pro‐proliferative effect is not restricted to monolayer culture (Figure 1I,J). Together, these results establish hPL as a more effective alternative to FBS for expanding hNPCs in both 2D and 3D settings.

### 3.2 hPL Facilitates NP Macromass Formation and Maintains Cellular Phenotype

**FIGURE 2:**
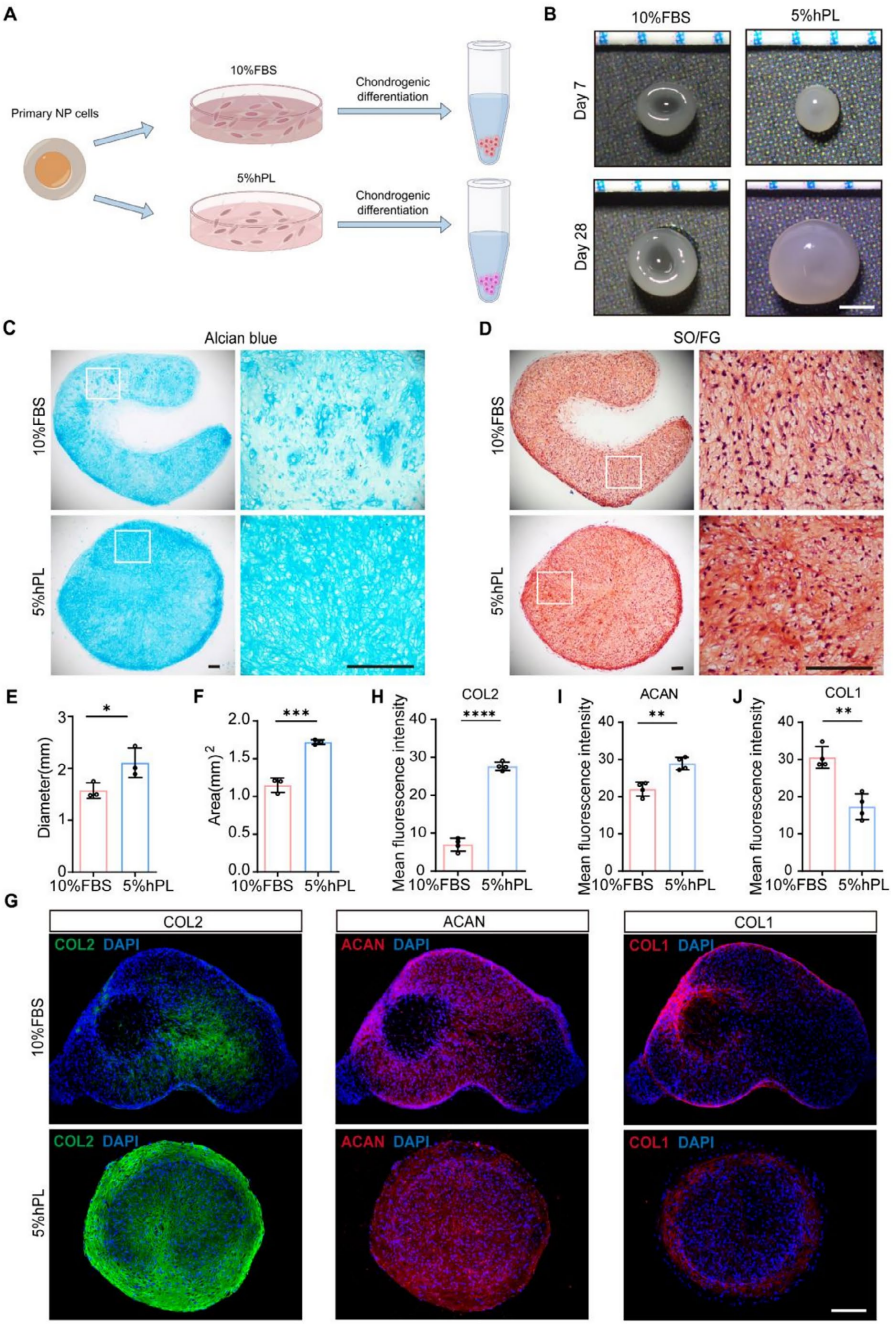
hPL enables the formation of large, ECM‐rich NP macromasses in 3D culture. (A) Schematic illustration of the protocol used to generate pellets. (B) Representative bright‐field images of tissues by FBS‐hNPCs and hPL‐hNPCs. Scale bar: 1 mm. (C, D) Representative images of Alcian blue and SO/FG staining of tissues. Scale bar: 100 μm. (E, F) Quantification of tissues diameter and cross‐sectional area on Day 28 (*n* = 3). (G) Representative immunofluorescence images of COL2, ACAN and COL1. Scale bar: 500 μm. (H‐J) Quantification of mean fluorescence intensity for COL2, ACAN and COL1 (*n* = 4). All data are presented as mean ± SD. *p < 0.05, **p < 0.01, ***p < 0.001, ****p < 0.0001, *p* values were determined using unpaired two‐tailed *t*‐test.

Given the superior expansion observed with hPL, we next assessed whether hPL‐hNPCs could form large functional tissue constructs suitable for clinical application using 3D pellet culture. Since 10% and 20% FBS produced similar results (Figure 1), we compared only 10% FBS and 5% hPL in subsequent experiments. Five hundred thousand hNPCs expanded in either hPL or 10% FBS were induced for 28 days to assess NP‐like functionality (Figure 2A). This induction protocol promotes the formation of aggregates [10, 27]. Following induction, cell aggregates formed in both groups. Notably, hPL‐hNPCs condensed into an intact disc‐like structure by Day 7 (Figure 2B), which enlarged over time, reaching a major axis of 2.11 ± 0.16 mm by Day 28 (Figure 2E). In contrast, FBS‐hNPCs showed reduced aggregation and formed a ring‐like structure on Day 7 (Figure 2B). Although tissue volume increased, these structures maintained their hollow morphology throughout the culture period, with a major axis of 1.58 ± 0.16 mm by Day 28 (Figure 2B,E). Quantitative analysis confirmed that tissue formed by hPL‐hNPCs possessed significantly larger areas than the FBS group (Figure 2F), demonstrating superior morphology and size.

ECM quality was then assessed by histological and immunofluorescence staining. Alcian blue and Safranin O/fast green (SO/FG) revealed glycosaminoglycan (GAG) deposition in both groups, though the distribution differed markedly: GAGs were uniformly distributed throughout hPL‐hNPC macromasses, while FBS‐hNPC constructs exhibited marked heterogeneity with areas of lower staining intensity (Figure 2C,D). Immunofluorescence analysis demonstrated that hPL‐hNPC macromasses displayed substantially enhanced COL2 deposition, with 3.97‐fold higher fluorescence intensity compared to FBS‐hNPCs (Figure 2G,H). ACAN expression was similarly increased in the hPL‐hNPCs group (Figure 2G,I). Importantly, COL1 expression was significantly lower in hPL‐hNPC macromasses, suggesting reduced progression toward a fibrotic phenotype (Figure 2G,J).

These results show that hPL‐hNPCs form larger, structurally intact macromasses with homogeneous ECM distribution and enhanced NP‐specific matrix deposition, in contrast to the smaller, hollow, and heterogeneous constructs seen in FBS culture.

### 3.3 hPL‐Derived NP Macromass Exhibit Superior Mechanical Properties

**FIGURE 3:**
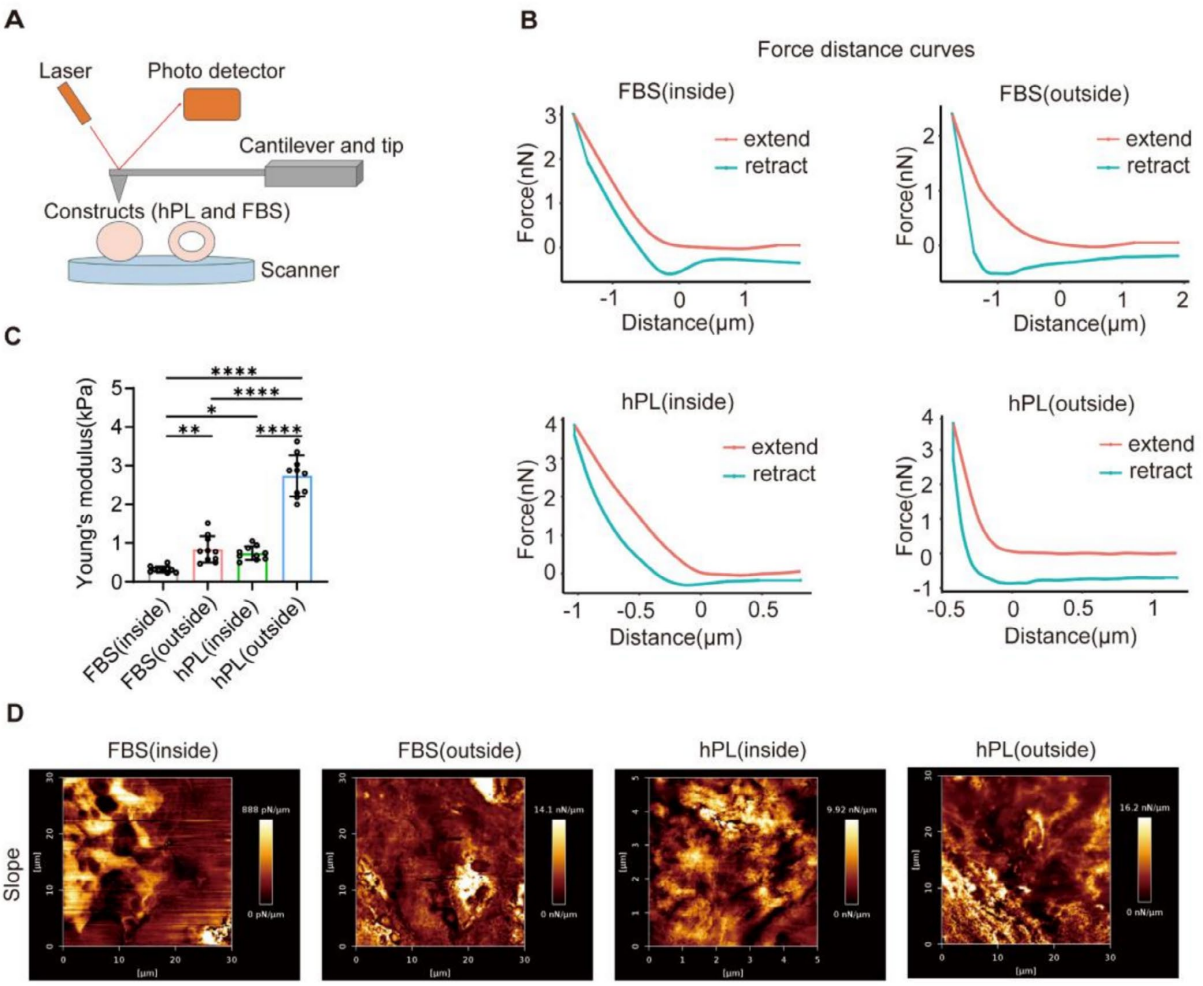
Macromass derived from hPL‐hNPCs exhibits enhanced mechanical properties. (A) Schematic of AFM indentation analysis on tissues by FBS‐hNPCs and hPL‐hNPCs. (B) Representative force distance curves obtained from different tissues. (C) Quantification of Young's modulus in inner and outer regions of tissues (*n* = 10 tests/region). (D) Representative AFM stiffness mapping images. All data are presented as mean ± SD. *p < 0.05, **p < 0.01, ****p < 0.0001, *p* values were determined using one‐way ANOVA followed by Dunnett's multiple comparison test.

To further evaluate the advantages of hPL‐derived NP macromass, we assessed its biomechanical properties using AFM (Figure 3A). Representative force distance curves demonstrated that hPL‐derived macromasses (hPL group) exhibited higher maximum forces compared to FBS‐derived constructs (FBS group), along with notable adhesion forces during retraction (Figure 3B). Young's modulus varied markedly across regions and culture conditions (Figure 3C). FBS group showed lower stiffness in the inner region (0.32 ± 0.08 kPa), whereas the outer region was substantially stiffer (0.84 ± 0.34 kPa, *p* < 0.01). hPL group exhibited significantly higher Young's modulus values overall. The inner region surpassed that of the FBS group (0.74 ± 0.18 kPa, *p* < 0.05). The outer region demonstrated a 2.7‐fold increase over the FBS group outer region and a 5.4‐fold increase compared to the FBS group inner region (*p* < 0.0001). Representative stiffness mapping images further confirmed more uniform stiffness distribution in hPL group compared to the heterogeneous pattern in FBS group (Figure 3D).

### 3.4 hPL‐hNPCs Exhibit Superior In Vivo Survival and NP Phenotype Retention

**FIGURE 4:**
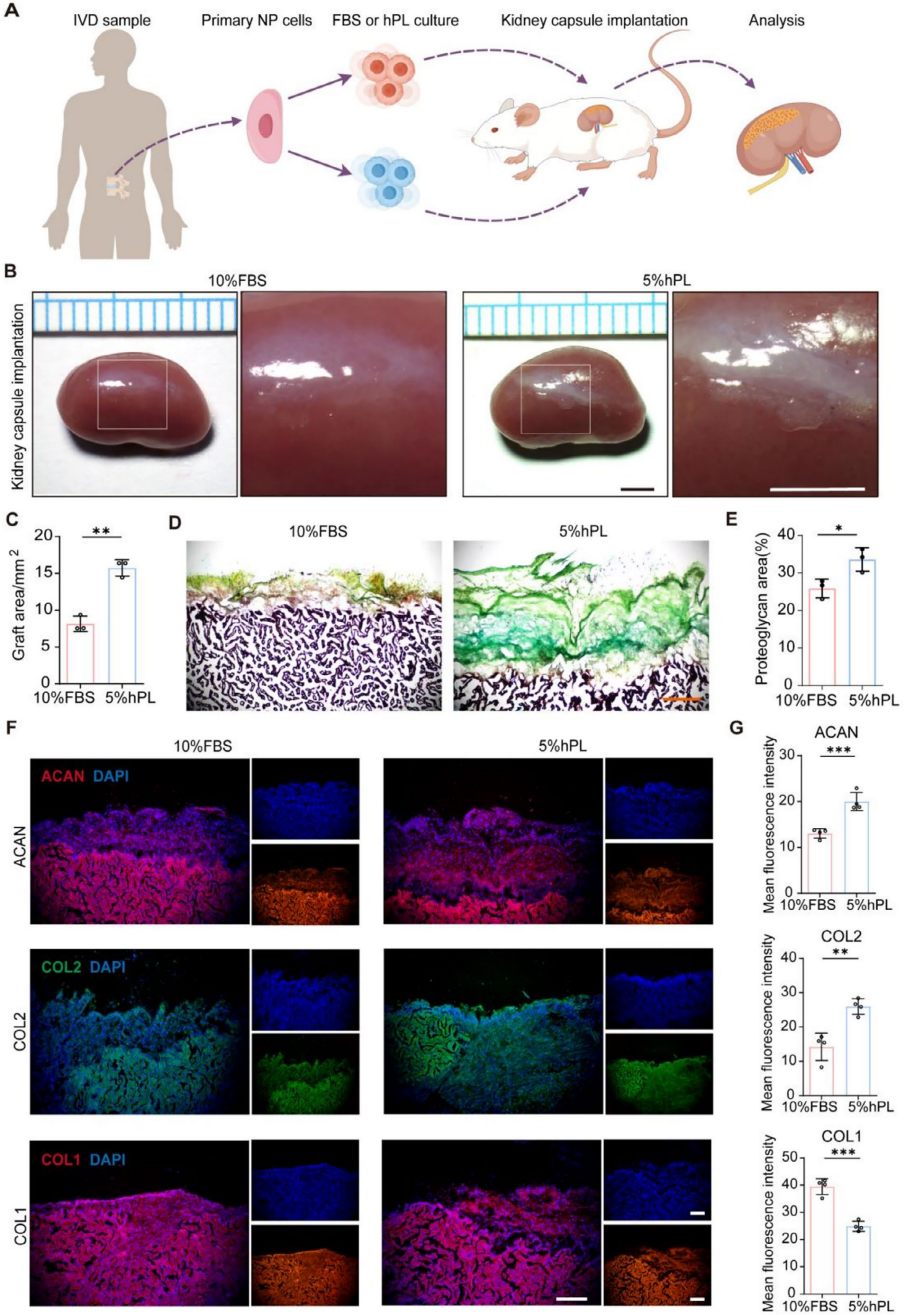
hPL‐hNPCs demonstrate superior graft viability and NP phenotype maintenance in vivo. (A) Schematic of the experimental design. (B) Representative macroscopic images of grafts at 12 weeks post‐transplantation. Scale bar: 2.5 mm. (C) Quantification of the relative graft areas (*n* = 3). (D) Representative images of Movat pentachrome staining of grafts. Scale bar: 200 μm. (E) Quantification of the relative area of blue‐stained regions in the grafts (*n* = 3). (F) Representative immunofluorescence images of ACAN, COL2 and COL1. Scale bar: 200 μm. (G) Quantification of mean fluorescence intensity (*n* = 4). All data are presented as mean ± SD. *p < 0.05, **p < 0.01, ***p < 0.001, *p* values were determined using unpaired two‐tailed t‐test.

To determine whether the in vitro advantages of hPL‐hNPCs translate to in vivo performance, hNPCs expanded in either FBS or hPL were embedded in Matrigel and implanted beneath the renal capsule of NPG mice to assess their survival, tissue‐forming capacity, and phenotype maintenance in a complex living environment [28]. After 12 weeks, grafts were harvested and analysed (Figure 4A). Macroscopic observation revealed significant differences in graft size between groups. Grafts derived from hPL‐hNPCs (hPL‐Grafts) were notably larger and more compact than those from FBS‐hNPCs (FBS‐Grafts) (Figure 4B,C), indicating greater in vivo tissue‐forming capacity.

To assess graft quality, we performed Movat Pentachrome staining. hPL‐Grafts displayed abundant blue‐stained areas and dense ECM (Figure 4D,E). In contrast, FBS‐Grafts exhibited sparse matrix with minimal ECM deposition (Figure 4D,E). To confirm the ECM proteins expression, we performed immunofluorescence staining (Figure 4F). Quantitative analysis confirmed that hPL‐Grafts expressed markedly higher levels of ACAN and COL2, while exhibiting reduced COL1 expression compared to FBS‐Grafts (Figure 4G), indicating maintained NP‐specific phenotype in vivo. These findings collectively demonstrate that hPL‐hNPCs possess superior tissue‐forming capacity and maintained NP phenotype in a complex living environment.

### 3.5 hPL Enhances hNPC Function Through Activation of Fatty Acid Oxidation

**FIGURE 5:**
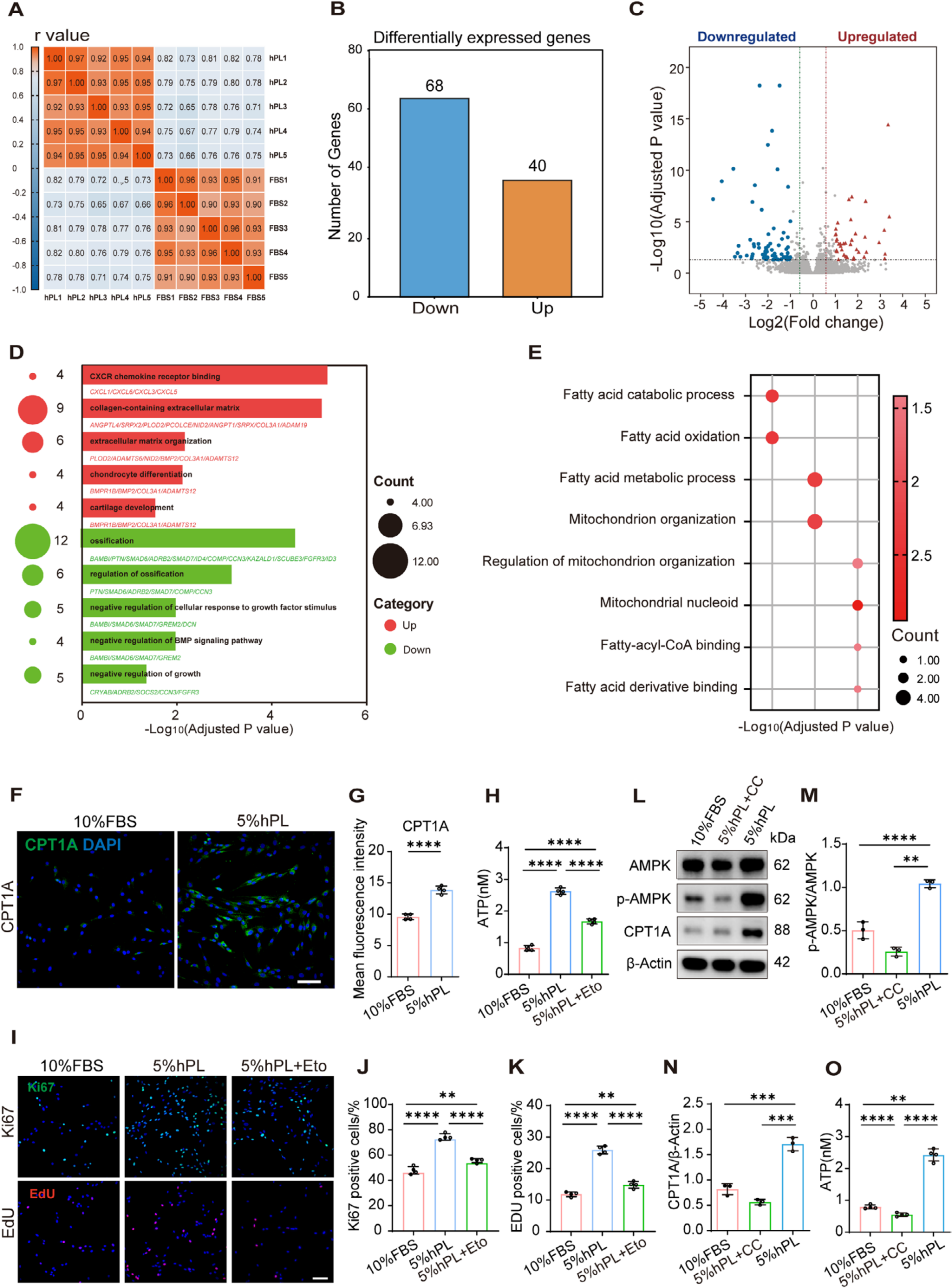
hpL enhances hNPC function via activation of mitochondrial FAO. (A) Heatmap of Pearson correlation coefficients among hNPC samples (*n* = 5 per group), demonstrating intra‐group reproducibility and separation between hPL and FBS groups. (B) Bar plot summarizing the number of differentially expressed genes (DEGs). (C) Volcano plot of DEGs between hPL‐ and FBS‐hNPCs. (D) Bar plot of Gene Ontology (GO) biological process enrichment analysis for DEGs. (E) GO enrichment analysis of mitochondria‐related differentially expressed genes. ‘Fatty acid catabolic process’ and ‘Fatty acid β‐oxidation’ were the most significantly enriched biological processes. (F) Representative immunofluorescence images of CPT1A. Scale bar: 100 μm. (G) Quantification of CPT1A fluorescence intensity (*n* = 4). (H) ATP levels in hNPCs cultured with 10% FBS, 5% hPL, or 5% hPL + etomoxir (Eto) (*n* = 4). (I) Representative immunofluorescence of Ki67 and EdU staining. Scale bar: 100 μm. (J, K) Quantification of Ki67^+^ and EdU^+^ cells. (*n* = 4). (L) Western blot images showing protein expression levels of p‐AMPK, total AMPK and CPT1A in hNPCs cultured with 10% FBS, 5% hPL + Compound C (CC), or 5% hPL. (M) Quantification of p‐AMPK/AMPK ratios (*n* = 3). (N) Quantification of CPT1A protein expression levels normalized to β‐actin (*n* = 3). (O) Quantitative analysis of ATP level in three groups of hNPCs following Compound C treatment (*n* = 3). All data are presented as mean ± SD. **p < 0.01, ***p < 0.001, ****p < 0.0001, *p* values were determined using unpaired two‐tailed *t*‐test or one‐way ANOVA.

To find the molecular mechanisms underlying the beneficial effects of hPL on hNPCs, bulk RNA sequencing was performed on cells expanded under either hPL or FBS conditions. Correlation heatmap analysis revealed high intra‐group consistency across biological replicates, with clear segregation between the hPL and FBS groups (*n* = 5 per group) (Figure 5A). Differential expression analysis identified 108 differentially expressed genes (log_2_(fold change) ≥ 1, adjusted *p*‐value ≤ 0.05) in hPL‐hNPCs compared to FBS‐hNPCs [29](Figure 5B). Among these, 40 genes were upregulated, including key regulators of cartilage formation and chondrogenic differentiation such as *BMP2, COL3A1* and *BMPR1B* [30, 31, 32]. Conversely, 68 genes were downregulated, notably including *FGFR3, SMAD6* and *SMAD7*, which are known inhibitors of cartilage proliferation and BMP signalling [33, 34] (Figure 5C). Gene Ontology (GO) enrichment analysis revealed that upregulated genes were predominantly associated with collagen‐containing extracellular matrix, chondrocyte differentiation and cartilage development [35]. In contrast, downregulated genes were enriched in biological processes related to ossification and negative regulation of the BMP signalling pathway (Figure 5D). Given that mitochondria play a crucial role in chondrogenic differentiation and energy metabolism [26, 36, 37, 38], we analysed mitochondria‐associated genes within our dataset. This analysis identified the top four enriched biological processes: fatty acid catabolic process, fatty acid β‐oxidation, fatty acid metabolic process and mitochondrial organization (Figure 5E).

As fatty acid oxidation (FAO) emerged as a prominently enriched pathway, we examined its functional relevance in hPL‐hNPC enhancement, carnitine palmitoyltransferase 1A (CPT1A), the rate‐limiting enzyme governing long‐chain fatty acid entry into mitochondria [39, 40], showed elevated expression in hPL‐hNPCs by immunofluorescence, confirmed by quantitative analysis of fluorescence intensity (Figure 5F,G). To determine whether enhanced FAO underlies the functional improvements in hPL‐hNPCs, FAO was inhibited using etomoxir, a specific CPT1A inhibitor [41]. ATP quantification demonstrated that hPL‐hNPCs produced significantly more ATP compared to FBS‐hNPCs, consistent with enhanced mitochondrial oxidative metabolism. Etomoxir treatment (5% hPL + Eto) substantially reduced ATP levels in hPL‐hNPCs, confirming that this elevated energy production depends on CPT1A‐mediated FAO (Figure 5H). Both Ki67 and EdU assays confirmed higher proliferation in hPL‐hNPCs than FBS‐hNPCs (Ki67: 72.39% ± 1.29% vs. 45.93% ± 1.54%; EdU: 25.91% ± 0.62% vs. 11.88% ± 0.37%) and etomoxir treatment markedly suppressed this effect (Ki67: 53.64% ± 0.80%; EdU: 14.85% ± 0.53%) (Figure 5I–K).

To investigate the upstream mechanism by which hPL activates FAO, we applied Compound C, a selective AMPK inhibitor and examined protein expression by Western blot. In hPL‐hNPCs, Compound C treatment (5% hPL + CC) markedly decreased p‐AMPK levels relative to the 5% hPL group. Accordingly, CPT1A protein expression was also reduced following AMPK inhibition, suggesting that hPL‐induced CPT1A upregulation is dependent on AMPK phosphorylation (Figure 5L–N). In line with this, ATP quantification showed that Compound C treatment significantly reduced ATP production in hPL‐hNPCs, a finding consistent with the results observed upon etomoxir treatment (Figure 5O). These data suggest that hPL activates AMPK‐CPT1A signalling to drive FAO‐dependent energy metabolism, supporting enhanced hNPC function.

### 3.6 Macromasses Generated From hPL‐hNPCs Facilitate IVD Repair in a Mouse Model

**FIGURE 6:**
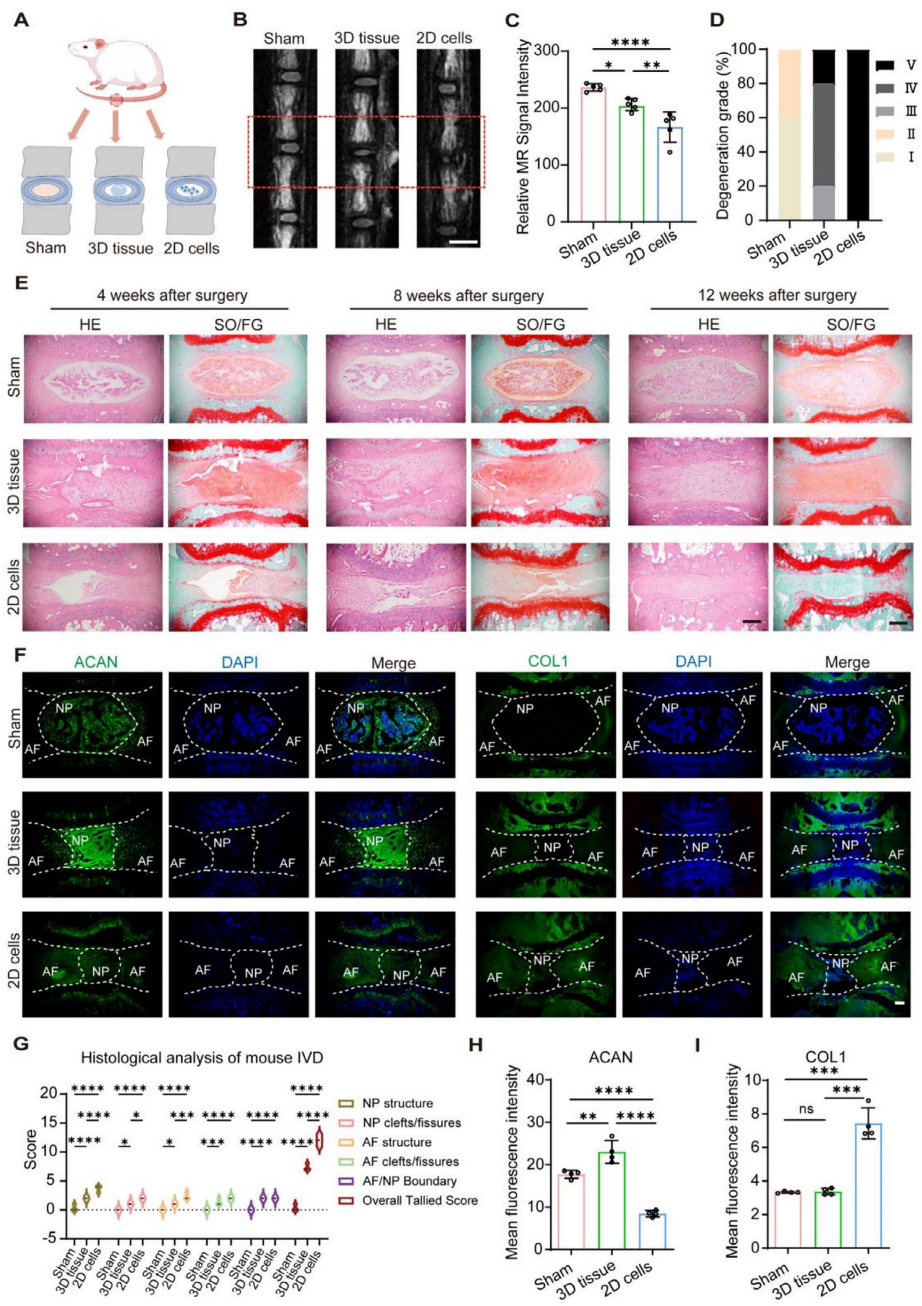
Macromass generated from hPL‐hNPCs promotes mouse IVD repair. (A) Schematic of IVD injury model and transplantation. (B) Representative MRI images of IVDs in each group at 12 weeks post‐surgery. Scale bar: 2 mm. (C) Quantification of high‐signal area ratio in MRI images (*n* = 5). (D) Pfirrmann grading assessment in each group (*n* = 5). (E) Representative histological images (H&E and SO/FG staining) at 4, 8 and 12 weeks post‐surgery. Scale bar: 200 μm. (F) Representative immunofluorescence staining images of ACAN and COL1 in the NP region of each group at 12 weeks post‐surgery. Scale bar: 100 μm. (G) Histological scoring of IVDs at 12 weeks post‐operation using the mouse IVD scoring system (*n* = 3). (H, I) Quantitative analysis of ACAN and COL1 in the NP region of each group (*n* = 4). All data are presented as mean ± SD. *p < 0.05, **p < 0.01, ***p < 0.001, ****p < 0.0001, *p* values were determined using one‐way ANOVA followed by Dunnett's multiple comparison test.

To evaluate the repair potential of macromasses generated from hPL‐hNPCs in vivo, we subsequently established an IVD injury model in 4‐week‐old NPG mice and transplanted the macromasses into the injured site (3D tissue group), with sham operation (sham group) and 2D hPL‐cultured cells transplantation (2D cells group) serving as controls (Figure 6A). At 12 weeks post‐operation, MRI signals in the 3D group were markedly better preserved than in the 2D group, though they did not fully recover to the level of the Sham group (Figure 6B,C). Pfirrmann grading was performed to quantitatively assess IVD degeneration. The sham group contained grade I–II. The 2D cell group exhibited grade V uniformly. In contrast, the 3D tissue group achieved grade III–V, demonstrating substantial improvement over the 2D cell group (Figure 6D).

Haematoxylin and eosin (H&E) staining at 4, 8 and 12 weeks post‐operation revealed that repaired IVDs in the 3D tissue group progressively developed more complete anatomical structures, with optimal restoration observed at 12 weeks (Figure 6E). SO/FG staining confirmed that the repaired IVDs in the 3D tissue group possessed GAG‐rich ECM. In contrast, the 2D cell group exhibited incomplete filling of the repair region, with lower GAG content (Figure 6E). To quantify the histological changes, we employed the mouse IVD histological scoring system, which evaluates NP structure, NP clefts/fissures, AF structure, AF clefts/fissures and AF/NP boundary, with higher scores indicating more severe degeneration [42]. At 12 weeks, histological scores showed a statistically significant difference between the 3D and 2D groups, as well as between the 3D and Sham groups, indicating that macromass transplantation significantly reduced IVD degeneration but was insufficient to restore the disc to a normal state (Figure 6G). Immunofluorescence staining was further performed to characterize changes in key ECM components (Figure 6F). ACAN expression in the NP region was higher in the 3D group than in the Sham group and significantly higher than in the 2D group. COL1 expression was significantly lower in both the 3D and Sham groups relative to the 2D group, with no statistically significant difference between the 3D and Sham groups (Figure 6H,I).

Collectively, these results demonstrate that macromass transplantation achieves better IVD repair than 2D hPL‐hNPCs injection, suggesting that 3D tissue organization substantially enhances the in vivo regenerative performance of hPL‐cultured hNPCs.

## 4 Discussion

**FIGURE 7:**
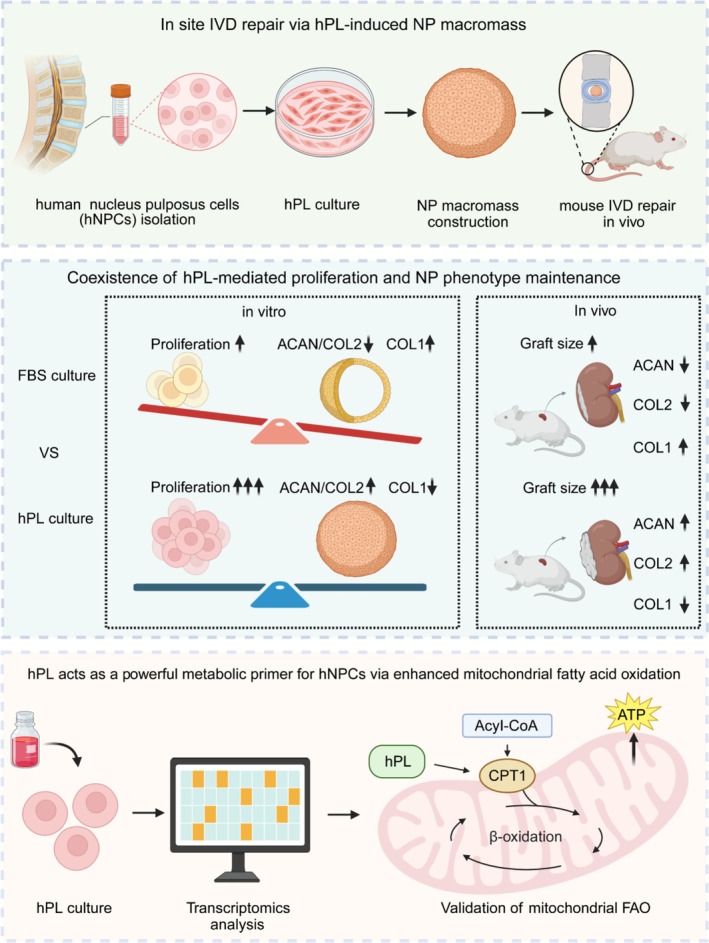
Overview of the study. Schematic illustrating that hPL serves as a metabolic primer, concurrently driving robust hNPC proliferation while preserving their native NP cellular phenotype. The hPL‐primed macromass has better mechanical properties and effectively repairs degenerated discs in situ.

The transplantation of NP analogs offers a promising treatment for IDD. Nevertheless, current strategies cannot produce large and functional human NP substitutes. The fundamental problem is the inability to expand cells while preserving their NP phenotype. Here, we demonstrate that hPL acts as a metabolic primer, enabling hNPCs to undergo extensive proliferation while retaining their NP characteristics. This metabolic enhancement facilitates the production of larger NP macromasses lacking necrotic cores, with improved mechanical properties and repair potential. Mechanistically, hPL upregulates mitochondrial FAO, thereby increasing ATP production that supports both cell expansion and phenotypic stability. This work establishes a novel strategy for IDD treatment through metabolic priming to generate functional NP substitutes (Figure 7).

The limited proliferative capacity of hNPCs in vitro represents a major obstacle to cell‐based disc regeneration strategies [43, 44]. We found that hPL culture substantially increases hNPC proliferation relative to conventional culture. hPL also promotes hNPC proliferation within 3D hydrogels, indicating its potential for scaffold‐based tissue engineering applications (Figure 1I,J). A key problem in traditional hNPC culture is phenotypic drift during expansion, marked by reduced expression of NP‐specific proteins and elevated expression of COL1 [45, 46]. This phenotypic instability severely compromises the regenerative capacity of expanded cells. The capacity for NP phenotype formation of hPL‐expanded hNPCs was validated in both NP macromass constructs and in vivo transplantation experiments. This phenotypic preservation is notable considering the high proliferation rate achieved concurrently, as these outcomes are typically mutually exclusive in cell cultures [47].

Beyond achieving adequate cell numbers, a more fundamental challenge in disc regeneration is the construction of high‐quality, functional NP analogs. The size limitation in 3D tissue constructs is due to metabolic constraints [48]. Cells in the center of large constructs face oxygen and nutrient diffusion barriers, typically leading to hypoxia and necrosis beyond 200 μm from the construct surface [49, 50, 51]. This diffusion limit explains why conventional pellet cultures rarely exceed 1–1.5 mm in diameter without developing necrotic cores [52]. Our data demonstrate that hPL‐hNPCs overcome this critical limitation, forming intact macromass tissues with an average diameter of 2.11 ± 0.16 mm without significant central necrosis (Figure 2). This represents a substantial advancement over conventional approaches that produce only small aggregates or hollow structures [10, 53]. Furthermore, renal capsule grafts formed by hPL‐hNPCs exhibited a significantly larger area than those formed by FBS‐hNPCs (Figure 4), demonstrating that hPL‐hNPCs maintain robust proliferative capacity even within the complex in vivo environment. These findings suggest that hPL provides not merely growth stimulation but creates a metabolic state conducive to sustained matrix synthesis throughout large tissue constructs. Taken together, these results hold important clinical implications. Notably, the absence of evident central necrosis in the NP macromass demonstrates that hPL enables hNPCs to develop exceptional hypoxic tolerance, an adaptation that renders them especially suited for engraftment and survival within the hypoxic NP microenvironment.

We identified that hPL‐induced metabolic shift toward mitochondrial FAO drives enhanced hNPC function. Mitochondria act as regulatory centers in cells, both generating energy and controlling signal transduction, metabolic balance and cell fate decisions [54, 55]. Recent studies show that hPL modulates mitochondrial function across different cell types. Chen et al. reported that a customized culture condition including hPL promotes robust mitochondrial biogenesis through AMPK pathway activation, achieving an 854‐fold increase in mitochondrial yield from human MSCs [26]. Du et al. demonstrated that hPL regulates MSC lineage commitment and paracrine functions through mitochondrial metabolic reprogramming [38]. Consistent with these reports, we found that hPL improves hNPC metabolism by enhancing mitochondrial FAO (Figure 5). This metabolic shift toward FAO matches the physiological requirements of hNPCs. While glycolysis generates only 2 ATP molecules per glucose molecule, FAO produces far more ATP per substrate unit [56, 57], supplying the energy needed for both rapid proliferation and matrix synthesis. Moreover, FAO has been shown to support stem cell maintenance and self‐renewal in various tissue contexts [39, 58], suggesting that hPL‐induced FAO activation may contribute not only to proliferative expansion but also to the preservation of progenitor‐like properties within the hNPC population. This metabolic shift holds significant physiological implications for enhancing transplanted cell survival and therapeutic efficacy. Native hNPCs reside in a distinctive microenvironment characterized by hypoxia, acidity, and nutrient deficiency [59]. In contrast, standard in vitro expansion under normoxic conditions (21% O_2_) and the inflammatory milieu encountered post‐transplantation can expose cells to supraphysiological oxidative stress, leading to mitochondrial dysfunction, cellular senescence and apoptosis [60, 61]. It is speculated that hPL‐induced metabolic shift toward FAO confers a protective advantage that directly addresses these challenges. Efficient and well‐coupled FAO reduces electron leak within the mitochondrial electron transport chain, thereby decreasing the primary generation of ROS [62, 63]. Given the degenerative disc environment, in which ROS overproduction is a well‐established driver of hNPC apoptosis, ECM degradation and accelerated senescence [64, 65], such metabolic resilience may be of particular therapeutic relevance. The hPL‐induced FAO preference may therefore confer a dual protective advantage: sustaining ATP supply under oxygen‐limited conditions while concurrently attenuating cellular oxidative damage. Such intrinsic metabolic resilience is critical for the long‐term persistence and efficacy of transplanted cells within the harsh environment of the degenerated disc [66, 67].

In conclusion, hPL provides metabolic priming to generate human NP macromass that exhibits enhanced mechanical properties and exceptional disc repair efficacy, achieving both rapid hNPC expansion and essential NP phenotype maintenance. Our study provides a new therapeutic strategy for IVD regeneration and enhances the mechanistic understanding of how to potentiate the innate regenerative capabilities of disc cells.

## Author Contributions

Peng Liu, Yibo Gan, Jian He and Yingbo Wang conceived and designed the work. Yingbo Wang and Ou Hu: performed hNPC isolation and in vitro experiments. Jun Zhu, Jungang Pu and Bo Hu performed NP tissue dissection and sample preparation. Yingbo Wang, Ou Hu, Sha Huang and Pulin Yan performed IHC and IF staining. Ou Hu, Yingbo Wang, Yangyang Li, Pulin Yan and Yibo Gan performed the animal model design and in vivo experiments. Jian Wu, Jian He, Yutong Wu and Peng Lin conducted the bioinformatics analysis. Yingbo Wang, Ou Hu and Sha Huang conducted the AFM testing and analysis. Yingbo Wang, Jian Wu and Ou Hu conducted the MRI in mice. Yingbo Wang, Ou Hu and Jian Wu were completed statistical analyses. Yingbo Wang, Jian Wu, Yibo Gan and Peng Liu drafted the initial manuscript.

## Funding

This work was supported by the National Natural Science Foundation of China (82430079, 32270887, 82272507, 32200654, 82430079 and 82472519), the National Key Research and Development Program of China (2022YFA1103202), the Chongqing High‐End Medical Talents for Middle‐aged and Young (YXGD202408), the Army Scientific and Technological Innovation Talents Prioritized Support Program (2023‐124), the Natural Science Foundation of Chongqing (CSTB2023NSCQ‐ZDJO008), Talent Recommendation Program of Army Medical Center (2025RCTJB03), the Project for Enhancing Innovation of Army Medical University (2023XJS39), the Chongqing Medical Leading Talent Project (YXLJ202508) and the Talent Innovation Training Program at the Army Medical Center (ZXZYTSYS09).

## Ethics Statement

This study was approved by the Ethics Committee of Daping Hospital, Army Medical University [Ethics Committee (2023‐48)]. Human nucleus pulposus tissue collection was conducted in accordance with the Declaration of Helsinki. All animal experiments were approved by the Institutional Animal Ethics Committee and performed in strict compliance with relevant guidelines.

## Conflicts of Interest

The authors declare no conflicts of interest.

## Supporting information


**Table S1:** A list of antibodies used for Western blot and immunofluorescence.

## Data Availability

The data that support the findings of this study are available from the corresponding author upon reasonable request.
