# Supplementary material for: Harnessing Metabolic Priming to Engineer Human Nucleus Pulposus Macromass Overcoming Scalability‐Phenotype Tradeoff
Source: Cell Prolif. 2026 Apr 28;59(9):e70215. doi: 10.1111/cpr.70215 (PMC13326001; doi:10.1111/cpr.70215)
Supplement: Supplementary file 1 [file CPR-59-e70215-s001.docx]

**Table S1: A list of antibodies used for Western blot and immunofluorescence**

| **Antibodies Name** | **Source** | **Catalog Number** | **Concentration** |
| --- | --- | --- | --- |
| Phalloidin | Abcam | ab176756 | 1:1000 |
| Ki-67 (D3B5) Rabbit mAb | CST | 9129T | 1:200 |
| Click-iT™ Plus EdU | invetrogen | C10640 | 1:200 |
| Aggrecan Polyclonal Antibody | Proteintech | 13880-1-AP | 1:100 |
| Collagen I/COL1A1 Rabbit mAb | Abclonal | A24112 | 1:100 |
| COL2A1 Antibody (M2139) | SANTA CRUZ | sc-52658 | 1:100 |
| CPT1A (E3Y1V) Rabbit mAb | CST | 97361T | 1:100 |
| Phospho-AMPK alpha (Thr172) Antibody | CST | 2535S | 1:1000 |
| AMPK alpha (D5A2) Rabbit Monoclonal Antibody | CST | 5831S | 1:1000 |
| beta Actin Recombinant Rabbit Monoclonal Antibody | HUABIO | HA722023 | 1:10000 |
| Goat Anti-Rabbit IgG H&L, HRP conjugated（bs-0295G-HRP） | Bioss | bs-0295G-HRP | 1:10000 |
| Goat anti-Mouse IgG (H+L) Alexa Fluor^TM^ 555 | Invitrogen | A32727 | 1:500 |
| Alexa Fluor^TM^ 488 goat anti-mouse IgG (H+L) | Invitrogen | A11001 | 1:500 |
| Alexa Fluor^TM^ 488 goat anti-rabbit IgG (H+L) | Invitrogen | A11008 | 1:500 |
| Donkey Anti-Rabbit IgG H&L (Alexa Fluor® 555) preadsorbed | abcam | ab150062 | 1:500 |
| Collagen I/COL1A1 Rabbit mAb | Abclonal | A22090 | 1:100 |
| COLⅡ Mouse Monoclonal (7F9) Antibody | AiFang biological | AF11387 | 1:100 |
